# Expression of HDAC3-Y298H Point Mutant in Medial Habenula Cholinergic Neurons Has No Effect on Cocaine-Induced Behaviors

**DOI:** 10.1523/ENEURO.0590-24.2025

**Published:** 2025-05-08

**Authors:** Vanessa Alizo Vera, Jessica E. Childs, Jisung Kim, Dina P. Matheos, Marcelo A. Wood

**Affiliations:** ^1^Department of Neurobiology and Behavior, University of California, Irvine, Irvine, California 92697; ^2^Center for the Neurobiology of Learning and Memory (CNLM), University of California, Irvine, Irvine, California 92697

**Keywords:** cocaine, conditioned place preference, extinction, hdac3, intravenous self-administration, medial habenula, reinstatement

## Abstract

Histone deacetylase 3 (HDAC3) is one of the most highly expressed HDACs in the brain shown to be a negative regulator of long-term memory formation. HDAC3 has also been shown to be involved in cocaine-associated behaviors, demonstrated by manipulations in the nucleus accumbens. Previous studies have demonstrated that expression of a dominant negative of a key HDAC3 target gene, nuclear receptor subfamily 4 group A member 2 (NR4A2), in cholinergic neurons of the medial habenula (MHb) blocked reinstatement of cocaine-induced conditioned place preference (CPP) as well as cue-induced intravenous self-administration (IVSA). Together, these findings suggested that HDAC3 would also be important for MHb-dependent reinstatement of CPP and IVSA, which we examined in this study. Contrary to our hypothesis, our results found that expression of an HDAC3 deacetylase dead point mutant within the cholinergic neurons of the mouse MHb had no effect on reinstatement or other cocaine-induced behaviors.

## Significance Statement

Understanding the molecular mechanism facilitating a persistent vulnerable state of relapse to drugs of abuse is critical for developing intervention programs. Epigenetic mechanisms hold promise for identifying novel mechanisms underlying relapse. However, in this study, we found no evidence for the role of HDAC3-Y298H, a deacetylase dead mutant, in medial habenula-dependent mechanisms of relapse-like behaviors.

## Introduction

Addiction, as defined by the National Institute on Drug Abuse, is a chronic neuropsychiatric disorder characterized by compulsive drug use despite adverse consequences, brief periods of abstinence, and an inevitable return to drug use (vulnerability to relapse). Drugs of abuse can dramatically alter brain states leading to long-lasting permanent changes in its functionality (Nestler, 2005). Vulnerability to relapse occurs, in part due to strong associations formed between environment and drugs of abuse resulting in abnormally resilient context-reward associated memories. Drug-associated contexts, cues, environments, or paraphernalia can precipitate relapse of drug-seeking behaviors. The persistent and long-lasting effects of drugs of abuse lead to vulnerability to relapse even after sustained periods of abstinence hinting at the enduring changes at the molecular level (Nestler and Lüscher, 2019).

A central mechanism potentially leading to persistent and long-lasting drug–associated changes is epigenetics (Malvaez et al., 2009, 2010; McQuown and Wood, 2010; Rogge et al., 2013; Rogge and Wood, 2013; White et al., 2016; Nestler and Lüscher, 2019). Epigenetic mechanisms can cause long-term changes to transcription patterns of a cell without changes to the DNA sequence. Histone deacetylases (HDACs) modulate transcription by deacetylating the lysine residues on the histone tails resulting in a tighter interaction between the DNA phosphate group and lysine residue. Histone deacetylase 3 (HDAC3) is a negative regulator of long-term memory processes (McQuown et al., 2011; Kwapis et al., 2017). Inhibition or deletion of HDAC3 can transform subthreshold learning events into long-term memory and also generate a form of long-term memory that persists beyond normal long-term memory (McQuown et al., 2011; Malvaez et al., 2013; Rogge et al., 2013).

HDAC3 has also been implicated in mechanisms of drug action. Focal deletion of HDAC3 in the nucleus accumbens can enhance cocaine–context-associated memories, leading to the hypothesis that HDAC3 acts as a negative regulator of long-term cocaine–context-associated memories (Rogge et al., 2013). Expression of an HDAC3 deacetylase dead mutant (Y298H) in D1R-expressing nucleus accumbens cells facilitated cocaine-induced conditioned place preference (CPP) and intravenous self-administration (IVSA; Campbell et al., 2021), while pharmacological inhibition during extinction created persistent extinction, preventing reinstatement (Malvaez et al., 2013). Together, these studies demonstrate a pivotal cell-type–specific role of HDAC3 in cocaine action.

In a recent study, a population of choline acetyltransferase (ChAT)-expressing neurons was identified in the ventral medial habenula (vMHb) as essential for cocaine-induced reinstatement of CPP and cue-induced reinstatement of drug seeking (via IVSA; López et al., 2018; Childs et al., 2024). Using c-Fos expression as a marker of neuronal activity, the MHb was found to be selectively engaged during cocaine-primed reinstatement. In addition, reinstatement led to an increase in histone 4 lysine 8 acetylation (H4K8Ac) in the vMHb, which is a substrate of HDAC3, suggesting that HDAC3 activity decreases during reinstatement, allowing for an increase in histone acetylation that facilitates gene expression necessary for reinstatement mechanisms. Chemogenetic activation of the MHb–ChAT population of neurons using ChAT-Cre mice was sufficient to induce reinstatement of CPP behavior in the absence of cocaine itself (López et al., 2018). These findings reveal that MHb–ChAT neurons may have a potential role in reinstatement of cocaine addiction, but perhaps not in the conditioning and extinction aspects of addiction models. Interestingly, cocaine-primed reinstatement driven by activation of MHb–ChAT neurons was found to be dependent on the expression/function of an HDAC3 target gene called nuclear receptor subfamily 4 group A member 2, *Nr4a2* (López et al., 2019; Childs et al., 2024). NR4A2 is critical for the development of dopamine cells (Perlmann and Wallén-Mackenzie, 2004), which hints to its importance in addiction. NR4A2's role in learning and memory has been extensively studied and found to be critical for activity-dependent long–term memory formation (Peña de Ortiz et al., 2000; Colón-Cesario et al., 2006; Hawk and Abel, 2011, 2012; McNulty et al., 2012). When an endogenous dominant-negative variant of NR4A2 was directly expressed in the ChAT neurons of the MHb, cocaine reinstatement was completely abolished in both CPP and IVSA (López et al., 2019; Childs et al., 2024).

We hypothesize that HDAC3 is a central regulator in transcription-dependent mechanisms underlying persistent drug-induced cellular and behavioral changes. We tested the hypothesis that HDAC3 within the MHb is a key regulator of cocaine-induced behaviors. The approach involved expressing a single amino acid mutation of HDAC3 that converts a tyrosine to a histidine (HDAC3-Y298H) in the cholinergic neurons of the MHb (via ChAT-Cre mice). The Y298H mutation has been shown to disrupt HDAC3 deacetylase activity (Kwapis et al., 2017). Contrary to our original hypothesis, we found no effects of the HDAC3-Y298H mutant expressed in ChAT–MHb cells on conditioning, extinction, or reinstatement of cocaine CPP and reinstatement of IVSA.

## Materials and Methods

All animal procedures were performed in accordance with the University of California, Irvine animal care committee's regulations. Single-housed male and female heterozygous ChAT-Cre mice (2–5 month old Jackson Laboratories 006410) were used for behavioral testing. Experiments were performed during the light cycle of a 12-h light/dark cycle, mice had ad libitum food and water access.

### Surgery

#### MHb infusion

Three weeks before behavior, 0.5 ml bilateral MHb infusions (M/L, ±0.35 mm; A/P, −1.5 mm; D/V, −3.0) of either AAV1-hSyn-DIO-GFP (GFP) or AAV1-hSyn-DIO-V5-HDAC3-Y298H were delivered stereotaxically using a 30 gauge Hamilton syringe (65459-01) and syringe pump (Harvard Apparatus Nanomite MA1 70-2217, 6 μl/h).

#### Jugular vein catheterization

One week before behavior, animals were implanted with an indwelling back-mounted jugular vein catheter for intravenous cocaine self-administration (see detailed methods in Childs et al., 2024). During surgery, the cannula was connected to a syringe of flushing solution (heparinized saline, 100 USP/ml in 0.9% saline and enrofloxacin) which prevented air embolism, irrigated the vein, and enabled blood draws to verify placement (dark red, draws easily and continuously). During 5–7 d of recovery, catheters were flushed daily to maintain catheter patency, which was verified before and after the self-administration period by observing a 5–10 s sedation after infusing the fast-acting anesthetic propofol (propofol sodium, Patterson Veterinary Supply). After recovery, animals were food restricted to 90% of presurgical weight over 3–4 d before the start of behavior. After a week of daily flushing, animals were handled.

#### Cocaine CPP

Following recovery from viral infusion, animals were subjected to CPP. Animals were handled for 2–3 min, during 5 consecutive days to reduce stress levels. Baseline preference levels were assessed by introducing mice into the chambers and recording the time spent in each for 15 min (pretest). Following pretest, animals were conditioned for 4 consecutive days receiving an injection of either cocaine–HCl (10 or 5 mg/kg, i.p.; Sigma-Aldrich) or 0.9% saline in a context-dependent manner. Afterward, animals received a posttest (in accordance with pretest) to assess the newly developed chamber preference. Preference was extinguished by reintroducing the mice to the chambers in a drug-free state for 15 min during 5 consecutive days. CPP training was finalized by reinstating the animals with a priming injection of cocaine–HCl (5 mg/kg, i.p.; Sigma-Aldrich).

#### Cocaine self-administration

Animals were allowed to self-administer cocaine in operant conditioning chambers (Med Associates) in 12 daily 1 h sessions. Most animals acquired self-administration within the first session; otherwise, on the second day (and third if needed), levers were baited with a drop of condensed milk. Failing to acquire self-administration or having a malfunctioning catheter was exclusion criteria. Animals advanced to an FR2 schedule after 4 d of successful FR1 response, which was defined as at least 10 active lever presses on Days 3 and 4. During self-administration, active lever presses elicited a cocaine reward (8.5 μg/kg/infusion) and a cue presentation (light/tone). There were no programmed consequences for inactive lever presses. Following self-administration, mice received a 30 d homecage withdrawal followed by a 5 h extinction session in which presses on the previously active lever were not rewarded or cued. Immediately after extinction, a 70 min cued reinstatement was induced by representing the drug-paired cues during the first 10 min. During reinstatement presses on the previously active lever resulted in cue presentation but no reward. Self-administration and extinction were analyzed using two-way repeated–measure ANOVA (Prism 10, GraphPad Software). Reinstatement data were analyzed using a one-way ANOVA. *P* values of <0.05 were considered significant.

#### Immunohistochemistry

**Figure 1.**
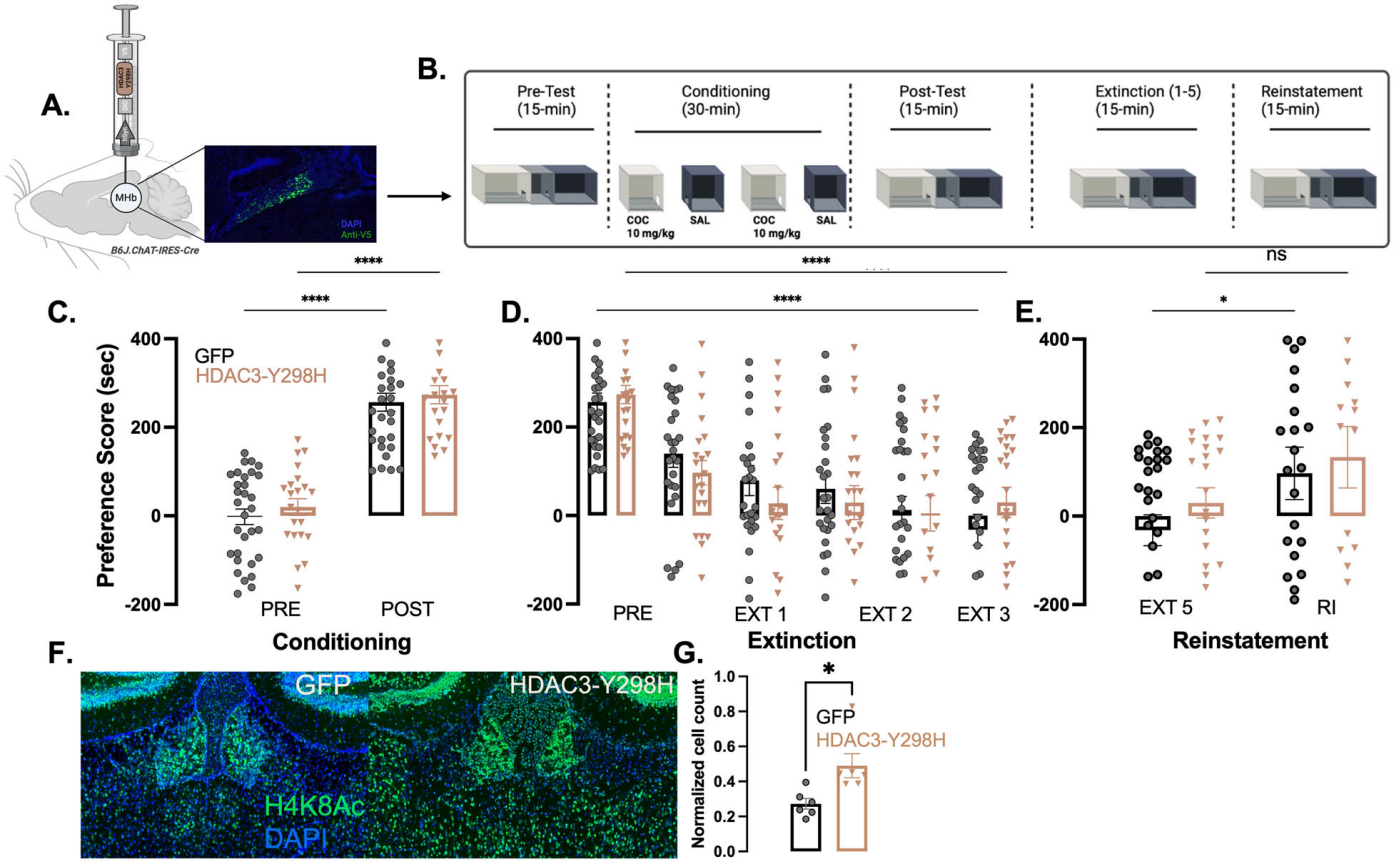
HDAC-Y298H has no effect on reinstatement of cocaine CPP. ***A***, Schematic of HDAC3-Y298H viral delivery to MHb and expression. Protein expression, fused V5 tag (green) compared with DAPI (blue). ***B***, Experimental timeline. After receiving viral infusion animals were conditioned for cocaine in CPP over 4 consecutive days, receiving cocaine–HCl (10 mg/kg, i.p.; Sigma-Aldrich) or 0.9% saline in a context-dependent manner. Following conditioning animal extinction their preference by reintroduction to the chambers without the presence of cocaine. Lastly, animals undergo reinstatement by receiving a priming infusion of 5 mg/kg of cocaine. ***C***, Cocaine CPP followed by extinction. Data analyzed using two-way repeated–measure ANOVA. Error bars indicate SEM. GFP males *n* = 22; females *n* = 8; HDAC3-Y298H males *n* = 19; females *n* = 3. ****p < 0.0001. ***D***, Five day extinction sessions. Data analyzed using two-way repeated–measure ANOVA. Error bars indicate SEM. ****p < 0.0001. ***E***, Cocaine-primed reinstatement. Data analyzed using two-way repeated–measure ANOVA. Error bars indicate SEM. *p < 0.01. ***F***, HDAC3-Y298H increases Histone 4 lysine 8 residue acetylation following reinstatement of cocaine CPP. Representative image of IHC against H4K8Ac in the MHb (left) GFP animals and (right) HDAC3-Y298H mice. ***G***, Animals with HDAC3-Y298H point mutation (*n* = 6) show an increase in immunoreactivity for H4k8Ac in the MHb compared with animals that received GFP control virus (*n* = 6; two-tailed *t* test; *t*_(6.8)_ = 2.89; *p* = 0.0238). Figure contributions: Vanessa Alizo performed the experiment and analyzed the data.

**Figure 2.**
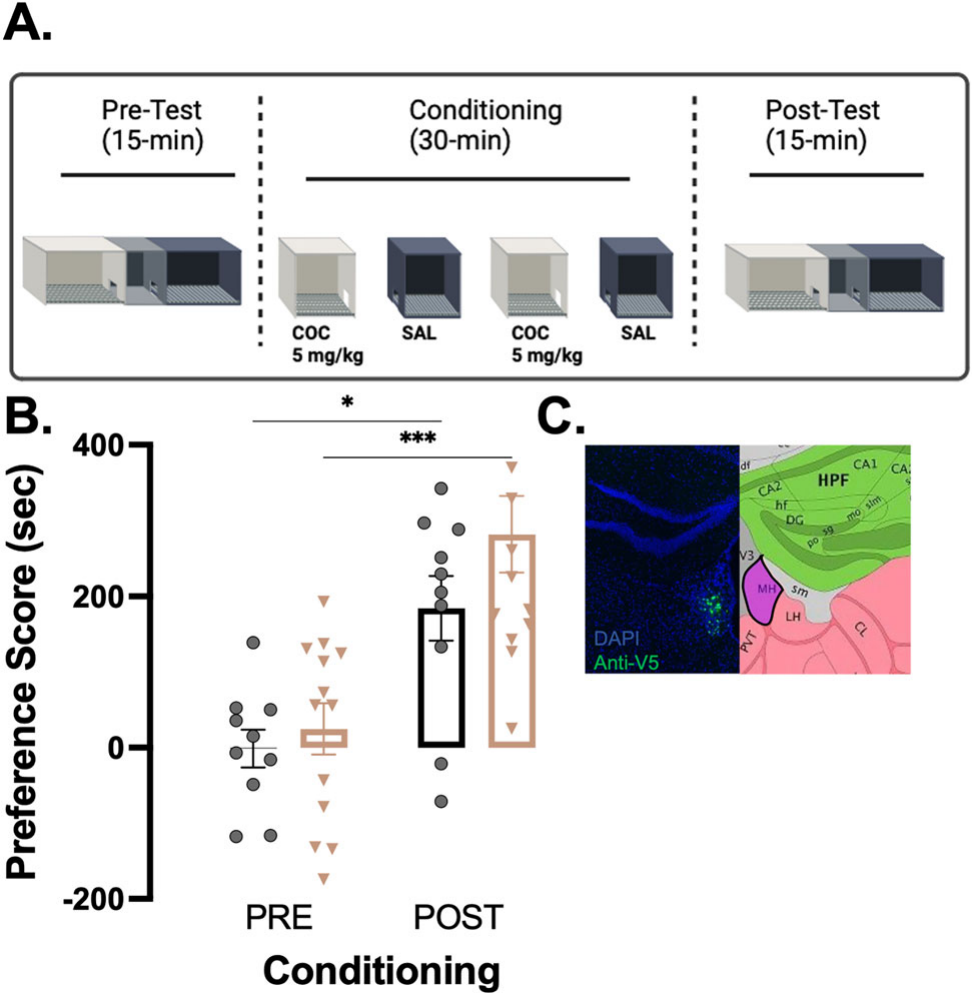
Expression of HDAC3-Y298H point mutation in cholinergic MHb neurons has no effect on 5 mg/kg cocaine CPP. ***A***, Experimental timeline. After receiving viral infusion, animals were conditioned for cocaine in CPP over 4 consecutive days, receiving cocaine–HCl (5 mg/kg, i.p.; Sigma-Aldrich) or 0.9% saline in a context-dependent CPP. ***B***, Cocaine CPP. Data analyzed using two-way repeated–measure ANOVA. Error bars indicate SEM. GFP males *n* = 6; females *n* = 4; HDAC3-Y298H males *n* = 6; females *n* = 7. **p < 0.001. ***C***, Protein expression, fused V5 tag (green) compared with DAPI (blue). Figure contributions: Vanessa Alizo performed the experiment and analyzed the data.

**Figure 3.**
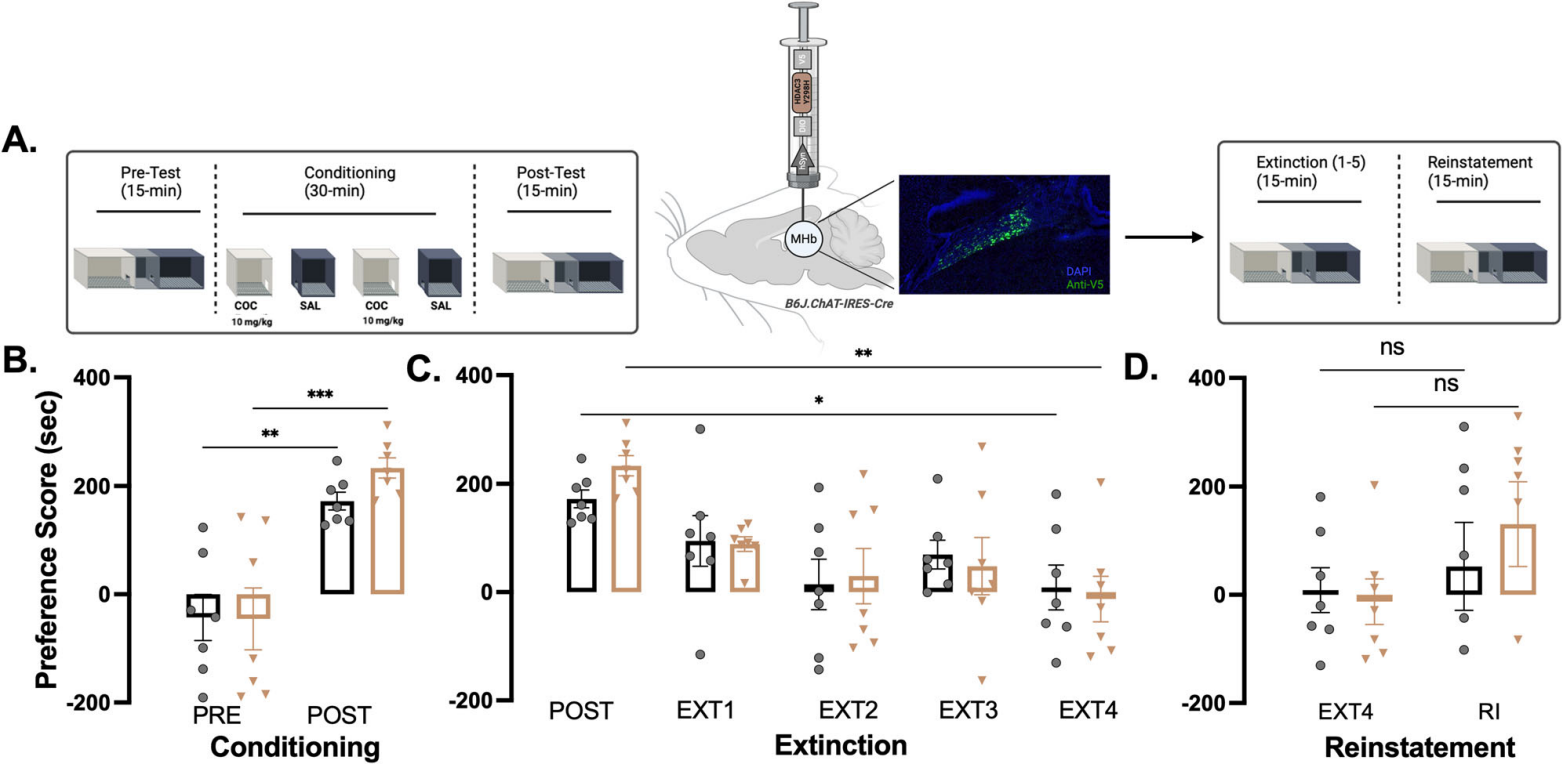
Expression of HDAC3-Y298H point mutation in cholinergic MHb neurons has no effect on extinction of cocaine CPP. ***A***, Experimental timeline and schematic of HDAC3-Y298H viral delivery to MHb and expression. Protein expression, fused V5 tag (green) compared with DAPI (blue). Animals were conditioned for cocaine CPP over 4 consecutive days, receiving cocaine–HCl (10 mg/kg, i.p.; Sigma-Aldrich) or 0.9% saline in a context-dependent manner. Following conditioning, the animal received a viral infusion ensued by extinction training. Lastly, animals underwent reinstatement by receiving a priming infusion of 5 mg/kg of cocaine. ***B***, Cocaine CPP followed by extinction training. Data analyzed using two-way repeated–measure ANOVA. Error bars indicate SEM. GFP males *n* = 4; females *n* = 3; HDAC3-Y298H males *n* = 4; females *n* = 3. **p < 0.01. ***C***, Five day extinction sessions. Data analyzed using two-way repeated–measure ANOVA. Error bars indicate SEM. **p < 0.01. ***D***, Cocaine-primed reinstatement. Data analyzed using two-way repeated–measure ANOVA. Error bars indicate SEM. **p < 0.01. Figure contributions: Vanessa Alizo performed the experiment and analyzed the data.

To visualize the spread of the HDAC3-Y298H-V5 viral expression, we performed immunohistochemistry with anti-V5 antibodies (Figs. 1–3). To examine the effect on histone acetylation, we examined H4K8Ac (Fig. 1*F–G*) from a subset of behavioral animals from Experiment 1. MHb coronal slices (20 μm, Leica CM 1850) were fixed with 4% paraformaldehyde, followed by three 1× PBS washes and 1 h block (8% normal goat serums, 0.01% Triton X-100, in 1× PBS). Blocking was followed by overnight primary antibody incubation (anti-V5 1:500 in block, Abcam; anti-histone H4K8Ac 1:500 in block, Abcam). Primary antibody was washed with two 1× PBS washes, followed by incubation with secondary antibody (1:250 in block, Alexa Fluor goat anti-rabbit 488) for 2 h at room temperature. A 15 min DAPI (1:10,000 in PB, Invitrogen) incubation was used to provide a nuclear counterstain for slide scanner imaging. Slides were partially dried before being coverslipped (VECTASHIELD) and sealed. Fluorescence images were taken on an Olympus slide scanner at 20× magnification. ImageJ was used for H4K8Ac positive cell quantification (Schneider et al., 2011).

### Quantification and statistical analyses

All statistical analysis and quantification were run using Graphpad Prism 10. Behavioral data were analyzed using two-way repeated–measure ANOVA or a one-way ANOVA for both self-administration and CPP. All data are presented with mean ± SEM.

## Results

### HDAC3-Y298H point mutation in cholinergic MHb neurons has no effect on reinstatement of cocaine CPP

To examine the role of HDAC3 in the cholinergic neurons of the MHb, we used a point mutant (Y298H) that abolishes deacetylase activity (Lahm et al., 2007) yet retains protein–protein interactions. Usage of this point mutant in previous studies demonstrated that HDAC3-Y298H results in impaired deacetylase activity (Kwapis et al., 2017), enhances histone acetylation (Kwapis et al., 2017; Malvaez et al., 2018), enhances synaptic plasticity (Campbell et al., 2021), enables long-term memory formation following subthreshold learning (Alaghband et al., 2017), and enhances long-term memory (Kwapis et al., 2017). Here, we tested the hypothesis that HDAC3 within the MHb acts as a negative regulator of cocaine-primed reinstatement of CPP. Thus, we predicted that the HDAC3-Y298H mutant would enhance reinstatement. In the first experiment, AAV expressing either DIO-HDAC3-Y298H-V5 or DIO-GFP was delivered into the MHb of ChAT-Cre male and female mice (Fig. 1*A*) 3 weeks prior to CPP (Fig. 1*B*). We found a main effect of conditioning, but no main effect of viral manipulation (Fig. 1*C*; cocaine 10 mg/kg, main effect of conditioning, *F*_(1,50)_ = 229; *p* < 0.0001; no main effect of virus, *F*_(1,51)_ = 0.8125; *p* = 0.3716; no conditioning × virus interaction, *F*_(1,50)_ = 0.01658; *p* = 0.8981). We found no viral dependent effect on extinction (Fig. 1*D*; main effect of behavior, *F*_(5,250)_ = 38.10; *p* < 0.0001; no main effect of virus *F*_(1,50)_ = 0.066; *p* = 0.7983; no behavior × virus interaction, *F*_(5,250)_ = 1.770; *p* = 0.1196). We also observed no effect of HDAC3-Y298H on cocaine reinstatement (Fig. 1*E*; main effect of behavior, *F*_(1,50)_ = 9.514; *p* = 0.0033; no main effect of virus, *F*_(1,50)_ = 0.6012; *p* = 0.4418; no behavior × virus interaction, *F*_(1,50)_ = 0.1122; *p* = 0.7390). HDAC3-Y298H-V5 viral expression was confirmed by IHC (Fig. 1*A*). Animals expressing the HDAC3-Y298H mutation display an increase in immunoreactivity for H4k8Ac in the MHb compared with animals that received GFP control virus (*t*_(6.8)_ = 2.89; *p* = 0.0238; Fig. 1*F*,*G*). Thus, HDAC3-Y298H expression had no effect overall on various stages of CPP. One caveat to this is that the point mutant was expressed prior to conditioning, which may alter downstream behavioral effects. Thus, the next experiments isolated just the conditioning phase and extinction phase.

### HDAC3-Y298H point mutation in cholinergic MHb neurons has no effect on conditioning of cocaine CPP

In Figure 1, we did not observe an effect on initial conditioning, but it may have been because of a performance ceiling as we used 10 mg/kg cocaine–HCl. Thus, in the next experiment to just examine the conditioning phase (Fig. 2*A*), we lowered the cocaine–HCl dose to 5 mg/kg, which has been used in the past to be able to better observe increases or decreases in performance (Campbell et al., 2021). Male and female ChAT-Cre mice received AAV-DIO-HDAC3-Y298H-V5 or AAV-DIO-GFP in the MHb 3 weeks prior to conditioning. With a 5 mg/kg cocaine–HCl conditioning dose, we found HDAC3-Y298H had no significant effect on conditioning (Fig. 2*B*; cocaine 5 mg/kg: main effect of conditioning, *F*_(1,21)_ = 30.94; *p* < 0.0001; no main effect of virus, *F*_(1,21)_ = 2.144; *p* = 0.1579; no conditioning × virus interaction, *F*_(1,21)_ = 0.8112; *p* = 0.3780). Cre-dependent overexpression of HDAC3-Y298H-V5 in the MHb Chat-Cre mice was confirmed using IHC (Fig. 2*C*). Thus, the HDAC3-Y298H mutant does not appear to affect the conditioning phase.

### HDAC3-Y298H point mutation in cholinergic MHb neurons has no effect on extinction of cocaine CPP

In past studies, it has been shown that HDAC3 inhibition (via pharmacological approach) facilitates extinction (Malvaez et al., 2013). To determine whether the HDAC3-Y298H mutant has an effect on extinction, we conditioned the animals prior to delivering AAV-DIO-HDAC3-Y298H-V5 or AAV-DIO-GFP (Fig. 3*A*). Virus was allowed to express 3 weeks prior extinction training (Fig. 3*B*). We found that HDAC3-Y298H had no effect on extinction (Fig. 3*C*; cocaine 10 mg/kg, main effect of conditioning, *F*_(3.281,39.38)_ = 12.67; *p* < 0.0001; no main effect of virus, *F*_(1,12)_ = 0.02309; *p* = 0.8817; no conditioning × virus interaction, *F*_(4,48)_ = 0.5897; *p* = 0.6717). Thus, the HDAC3-Y298H mutant does not appear to affect the extinction phase.

### HDAC3-Y298H point mutation in cholinergic MHb neurons has no effect on cocaine seeking

**Figure 4.**
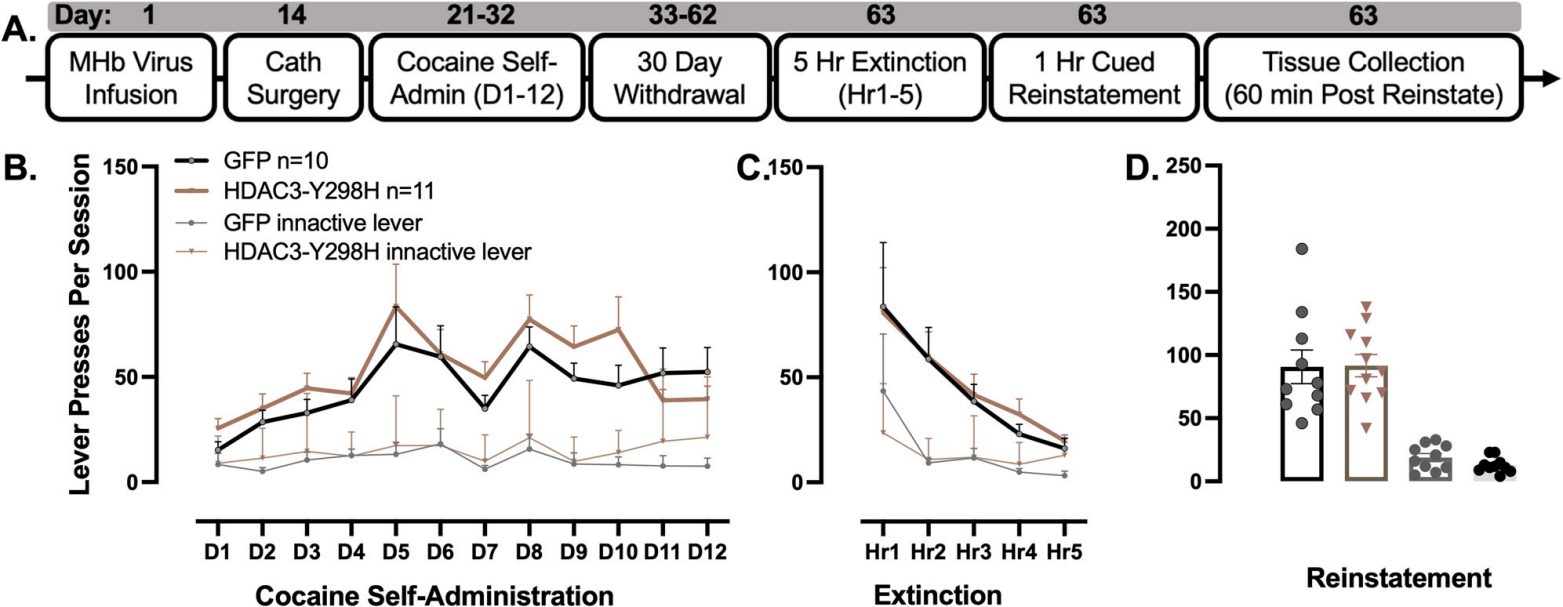
Expression of HDAC3-Y298H point mutation in cholinergic MHb neurons has no effect on cocaine seeking. ***A***, Experimental timeline. After receiving infusion and catheter surgeries, mice self-administered cocaine for 12 d in operant conditioning chambers (D1–12). During self-administration, active lever presses resulted in a cocaine reward (8.5 μg/kg/infusion) plus a tone/light cue presentation. After 12 d of self-administration, mice experienced a 30 d homecage withdrawal period. Mice were then extinguished in a single 5 h extinction session, in which lever presses were inconsequential. Reinstatement was then induced by exposing mice to the drug-paired cues for 10 min preceding a 1 h cued reinstatement session, during which lever presses elicited cues but no cocaine rewards. Animals were killed, and the tissue was collected 1 h after reinstatement. ***B***, Cocaine self-administration followed by 30 d homecage withdrawal. Data analyzed using two-way repeated–measure ANOVA. Error bars indicate SEM. GFP males *n* = 10; HDAC3-Y298H males *n* = 11. ***C***, Five-hour extinction session. Data analyzed using two-way repeated–measure ANOVA. Error bars indicate SEM. ***D***, Cued reinstatement. Data analyzed using one-way ANOVA. Figure contributions: Jessica Childs performed the experiment and Vanessa Alizo analyzed the data.

To examine the effects of HDAC3-Y298H-V5 within the ChAT neurons of the MHb during cocaine seeking, we used an IVSA model of relapse seeking (Fig. 4*A*). In this experiment, male ChAT-Cre mice received AAV-DIO-HDAC3-Y298H-V5 or AAV-DIO-GFP to the MHb. Three weeks later, mice were trained to self-administer cocaine (Fig. 4*A*), which was followed by a 30 d withdrawal period which, shown in previous studies, can elicit robust cue-induced reinstatement of drug seeking (Childs et al., 2024). During cocaine self-administration, presses on the active lever exceeded presses on the inactive lever in both groups (Fig. 4*B*; GFP, *F*_(1,18)_ = 33.02; *p* < 0.0001; HDAC3, *F*_(1,20)_ = 53.87; *p* < 0.0001). During self-administration, there were no differences between groups in response to the active lever (Fig. 4*B*; *F*_(1,19)_ = 1.355; *p* = 0.2588). After 30 d of homecage withdrawal, there were no differences between groups in extinction (Fig. 4*C*; *F*_(1,19)_ = 0.04528; *p* = 0.8338). At the end of extinction (E5), all animals received 10 min of cue priming to induce reinstatement, which was followed by a 1 h cued reinstatement session; behaviorally, there were no differences between groups (Fig. 4*D*; *t*_(19)_ = 0.05943; *p* = 0.9532). Thus, the HDAC3-Y298H mutant does not appear to affect cocaine–IVSA behaviors.

## Discussion

In this study, we investigated whether expression of an HDAC3 deacetylase dead point mutation (HDAC3-Y298H) within ChAT neurons of the MHb would enhance reinstatement in both the CPP and IVSA paradigms. In previous studies, it has been shown that HDAC3 is a key negative regulator of long-term memory formation such that deletion or inhibition of HDAC3 transforms a subthreshold learning event into long-term memory and generates a form of long-term memory that persists beyond normal memory (McQuown et al., 2011). HDAC3 deletion enhances memory processes in an NR4A2-dependent manner (McQuown et al., 2011), suggesting that NR4A2 is an important downstream effector gene of HDAC3 epigenetic transcriptional regulation. Earlier evidence demonstrated that NR4A2 function is necessary in MHb–ChAT neurons for reinstatement of cocaine-induced CPP and cue-induced IVSA (López et al., 2019; Childs et al., 2024). Thus, a logical hypothesis was that HDAC3 would have a role in MHb-dependent reinstatement. However, as our data show, we found that expression of the HDAC3-Y298H point mutant has no effect on reinstatement or even acquisition/consolidation and extinction of CPP and IVSA. It should be noted that given the negative results and in an attempt to reduce animal numbers, only males were used for the IVSA experiment.

This was a surprising result given that HDAC3 physical occupancy on the *Nr4a2* promoter has been observed via HDAC3-ChIP-qPCR in small tissue punches from the MHb (López et al., 2019). It is possible that a deletion of HDAC3, or a different point mutant, might have a larger impact on behavior. We have used the HDAC3-Y298H mutant in several past studies (as detailed in the first paragraph of the results section describing the use of this point mutant). However, a homozygous deletion of *Hdac3* or perhaps a different point mutant in HDAC3 would reveal the role of HDAC3 in MHb-dependent behaviors.

It is also possible that enzymes that counter HDAC3 deacetylase activity are not as active in the MHb as observed in learning and memory regions such as the hippocampus. For example, when HDAC3 is deleted or inhibited, there is a large and immediate increase in histone acetylation (McQuown et al., 2011; Kwapis et al., 2017). HDAC3 is a deacetylase, which means that the increase in histone acetylation is a result of active histone acetyltransferases (HATs) like CREB-binding protein. There is a constant and active counteraction between HDACs and HATs, such that disabling HDAC3 leads to immediate increase in histone acetylation, which in turn facilitates gene expression required for long-term memory processes. It is possible then that such a phenomenon (as observed in the hippocampus) may be weaker or not be as active in the MHb; however, Figure 1 shows that the point mutant led to an increase in H4K8Ac. Thus, it is not entirely clear why this particular point mutant of HDAC3 did not have an effect in any behavior we examined in this study.

In summary, this negative data presented here are important as it helps clarify the overall picture of the epigenetic and molecular mechanisms underlying MHb-dependent behaviors related to the action of drugs of abuse.

## Synthesis

Reviewing Editor: Laura Bradfield, University of Technology Sydney Decisions are customarily a result of the Reviewing Editor and the peer reviewers coming together and discussing their recommendations until a consensus is reached. When revisions are invited, a fact-based synthesis statement explaining their decision and outlining what is needed to prepare a revision will be listed below. The following reviewer(s) agreed to reveal their identity: Paul Kenny, David Dietz.

The reviewers agree that all their previous concerns have been addressed, and that the paper advances the field. Thank you for submitting to eNeuro.
